# PROLONGED MECHANICAL VENTILATION: CHARACTERISTICS OF PATIENTS TREATED AT HOME COMPARED TO HOSPITAL LONG-TERM CARE

**DOI:** 10.1093/geroni/igz038.1678

**Published:** 2019-11-08

**Authors:** Jeremy M Jacobs, Esther-Lee Marcus, Jochanan Stessman

**Affiliations:** 1 Dept of Geriatrics and Rehabilitation, Jerusalem Institute for Aging Research, Hadassah Hebrew University Hospital, and Clalit Health services, Jerusalem, Israel; 2 Head, Chronic Ventilator-dependent Division, Herzog Hospital, Jerusalem, Israel

## Abstract

Rising numbers of patients receiving Prolonged Mechanical Ventilation (PMV) pose a challenge, and advancing technology supports ventilators appropriate for either Home or Hospital Long Term Care (HLTC).Data guiding decisions concerning place of care are lacking. This study describes the characteristics of the majority (120/123) of all PMV patients aged ≥18 (and their caregivers) in Jerusalem, covered by the Clalit Health Service, treated either with Home Hospital or HLTC. Patients were more alert and communicative at Home vs. HLTC (40/46 vs. 22/74), younger (54 vs.73 years, p12 years (36% vs.21%, p=0.1), and without legal guardian (59% vs. 12%, p<0.01). Primary reason for PMV at home was degenerative neuromuscular disease (59% vs. 28%), compared to post resuscitation/sepsis/CVA in HLTC patients (17% vs. 62%), who suffered more comorbidity, functional decline post-PMV, and pressure sores (0% vs. 42%). Ventilation was more likely to be planned at home vs HLTC (33% vs. 8%), and yet 119/120 were without Advanced Directives prior to PMV. Caregivers at home tended to be spouses (48% vs. 31%) and offspring at HLTC (17% vs. 47%), with reduced Modified Caregiver Strain Index at home (10.5 vs. 12.9, p=0.12). Mortality during follow-up was lower at home (15.2% vs. 27%). Costs to the health fund for home versus HLTC were approximately 1:3. Our findings suggest that with appropriate targeting of eligible PMV patients, Home Hospital may be the preferred model of care for patients, caregivers and healthcare providers.

